# THE CURRENT STATUS OF ACADEMIC GERONTOLOGY PROGRAMS IN THE US

**DOI:** 10.1093/geroni/igae098.0817

**Published:** 2024-12-31

**Authors:** Judith Sugar

**Affiliations:** University of Nevada, Reno, Reno, Nevada, United States

## Abstract

With the growing population of older people in the U.S., it is more important than ever to increase the number of well-educated gerontology professionals. It is no surprise to GSA members that America’s aging population is projected to grow by leaps and bounds over the next several decades—reaching 82 million by 2050. Yet, the number of academic gerontology programs, and hence graduates from those programs, have not been keeping pace with the need for professionals with knowledge and skills in gerontology. This presentation uses recently compiled data to demonstrate that we are falling behind in our ability to educate a workforce competent to provide services for older people and their loved ones, and to positively impact older adults’ quality of life and their ability to make valuable contributions to our society. When compared with data from 20 years ago, we have had virtually no change in the number of associate, baccalaureate, or master’s degree programs, nor in the aggregated number (all levels) of certificates. The only category in which growth has occurred is in the number of undergraduate minors. The numbers, the needs for services, and the prospects for providing products and services for older adults and their loved ones offer more possibilities than ever before for professionals who are well-educated in Gerontology. It is time to re-energize the field of Gerontology to strengthen and grow our academic programs and recruit more students to take advantage of the opportunities and meet the challenges of our aging population.

